# Influence of the Casein Composite Genotype on Milk Quality and Coagulation Properties in the Endangered Agerolese Cattle Breed

**DOI:** 10.3390/ani10050892

**Published:** 2020-05-20

**Authors:** Sara Albarella, Maria Selvaggi, Emanuele D’Anza, Gianfranco Cosenza, Simonetta Caira, Andrea Scaloni, Annunziata Fontana, Vincenzo Peretti, Francesca Ciotola

**Affiliations:** 1 Department of Veterinary Medicine and Animal Production, University of Naples Federico II, via Delpino 1, 80137 Naples, Italy; emanuele.danza@unina.it (E.D.); vincenzo.peretti@unina.it (V.P.); francesca.ciotola@unina.it (F.C.); 2 Department of Agricultural and Environmental Science, University of Bari Aldo Moro, 70126 Bari, Italy; maria.selvaggi@uniba.it; 3 Department of Agriculture, University of Napoli Federico II, 80055 Portici, Italy; giacosen@unina.it; 4 Proteomics & Mass Spectrometry Laboratory, ISPAAM, National Research Council, 80147 Naples, Italy; simonetta.caira@ispaam.cnr.it (S.C.); andrea.scaloni@cnr.it (A.S.); 5 Laboratory of milk analyses (LSL), Italian Breeders Association (AIA), 00054 Maccarese, Italy; fontana.t@aia.it

**Keywords:** *CSN1S1*, *CSN2*, *CSN3*, *Bos taurus*, reological properties, milk traits

## Abstract

**Simple Summary:**

Characterization of variants in casein genes allows breeders and researchers to select the most suitable cows for milk production within the same breed. It has been observed that milk from different cattle breeds with the same casein composite genotype shows different chemical and coagulation properties. The aim of this work was to characterize *CSN1S1*, *CSN2* and *CSN3* gene variants in Agerolese cattle, an Italian autochthonous breed, milk of which is used to produce “Provolone del Monaco”, a PDO cheese with a relevant economic interest for the Lattari Mountains area and the Sorrento Peninsula (Naples, Italy). According to the results, Agerolese cattle population shows a low genetic variability and a high prevalence of the A^2^ allele of *CSN2*. As regard chemical composition and coagulation properties, BBA^1^A^2^AB, BBA^2^A^2^BB and BBA^2^A^2^AB composite genotypes showed the best parameters. These results will be used to promote the genetic value of Agerolese cattle and to help breeders to improve their breeding practice.

**Abstract:**

The aim of this study was the characterization of CSN1S1, CSN2 and CSN3 genetic variability in Agerolese cattle, and the investigation of the effect of casein composite genotypes (CSN1S1, CSN2 and CSN3) on quality and coagulation traits of the corresponding milk. To these purposes, blood and milk from 84 cows were sampled and analysed. Allele frequencies at *CSN2* and *CSN3* revealed no Hardy–Weinberg equilibrium in the population with a prevalence of allele A^2^ for *CSN2* and allele B for *CSN3*. BBA^1^A^2^AB and BBA^2^A^2^AB composite genotypes were the most common in the population. BBA^1^A^2^AB showed a higher total solids and fat content (12.70 ± 0.16 and 3.93 ± 0.10, respectively), while BBA^2^A^2^BB showed the best coagulation properties (RCT 12.62 ± 0.81; k_20_ 5.84 ± 0.37; a_30_ 23.72 ± 1.10). Interestingly, the A^2^ allele of *CSN2* was very widespread in the population; thus, it will be intriguing to verify if A^2^A^2^ Agerolese cattle milk and the derived cheese may have better nutraceutical characteristics.

## 1. Introduction

Agerolese cattle are a dual-purpose (dairy and beef) autochthonous breed reared in Southern Italy. It originated in the area of the Lattari Mountains and the Sorrento Peninsula (Naples, Italy) and underwent crossbreeding along successive generations with Bretonne, Brown Swiss, Jersey and Friesian breeds. It is a rustic breed that produces milk with excellent organoleptic characteristics, mainly destined to the production of the PDO (Protected Denomination of Origin) cheese (reg. CE no. 121/2010) “Provolone del Monaco”. This is a fine *pasta filata* cheese that has a melon or pear shape without the head divided in six sides and that can weight from 2.5–8 kg. The rind is yellow and almost smooth with inlets in correspondence of the cord used to hang it. The flavour is sweet and buttery with a spicy taste whose intensity depends on the length of seasoning which must be at least six months. A minimum of 20% of Agerolese milk must be used for its production [1]. “Provolone del Monaco” PDO cheese has become a driving force for the economy of the area occupying 30 breeders able to generate an annual turnover of 3 million euros. This has led to a renewed interest in reproducing this breed, thus preventing its extinction; to date there are 285 animals enrolled in the birth register (Italian Breeders Association—AIA). Nowadays, there is very little information about Agerolese cattle genetics, especially regarding the evolution of casein genes through the centuries as an effect of inbreeding. Selvaggi and coworkers observed an association between different genotypes at the *SIRT1* g.-274C>G locus and milk production performances and reproductive traits [2]. In other studies, a positive effect of the TT genotype of *STAT5A/Msl* I locus on fat and protein content of milk was observed, while no variability at *STAT5A/Ava* I locus was found [3], despite the common origin with the Podolica breed that shows high genetic variability at this locus [4,5]. From a cytogenetic point of view, Agerolese cattle exhibited very stable genome, when compared with the other Italian cattle breeds [6], and low incidence of chromosomal abnormalities [7]. There is a lack of knowledge concerning genetic variability in loci directly involved in milk production and clotting properties in Agerolese breed. 

The most studied milk proteins are α_s1_-CN, β-CN and κ-CN, which are encoded by *CSN1S1*, *CSN2* and *CSN3* genes, respectively. *CSN1S1 locus* shows nine alleles (A, B, C, D, E, F, G, H and I) that correspond to nine protein variants [7]. Only for the G variant, a considerable reduction of α_s1_-casein content in the milk has been reported, with a consequently lower milk coagulation times and curd firmness [8]; due to the reduction in the milk coagulation proprieties (MCP), the presence of the G variant has a negative impact on dairy productions [9]. β-CN constitutes up to 45% of the casein and 12 genetic variants (A^1^, A^2^, A^3^, B, C, D, E, F, G, H^1^, H^2^ and I) have been isolated and characterized, among which A^1^ and A^2^ are the most common [10]. β-casein variants play an important role in cheese yield and quality and their concentration is positively associated with good rennet properties of milk [11]. In particular, the *CSN2 B* allele is associated with an increase of milk casein content and smaller casein micelle size [12,13]. The κ-CN proportion in milk and its variants, namely A or B, are important because of their influence on clotting time, curd firming time and curd firmness [14,15]. In *Bos genus* the encoding gene, *CSN3*, is characterized by at least 14 alleles (A, A^I^, B, B^2^, C, D, E, F^1^, F^2^, G^1^, G^2^, H, I and J) coding 13 proteins and one synonymous (A^I^). In particular, allele *B* is associated with higher protein content, determines smaller average casein micelle sizes and better coagulating properties [16]. κ-CN is the milk component mainly influencing milk coagulation properties; in this context, it has been proven that the proportion of κ- and α-CN have positive effects on curd firming time and curd firmness [17]. Milk production traits and milk clotting properties (MCP) are relevant to the dairy industry as they relate to socio-economic factors affecting milk production. In order to evaluate MCP, three parameters have principally been used: rennet coagulation time (RCT, min), curd-firming rate (k_20_, min) and curd firmness (a_30_, mm) [16,18].

The effects of genetic variants of milk proteins on MCP have been thoroughly investigated in cattle; only in a few cases, the effect of polymorphisms at the casein gene cluster has been found to be associated with MCP. In order to manage and preserve endangered livestock breeds, the knowledge of their performances and genetic variability is essential. It is generally agreed that *B* variants of β-CN and κ-CN favourably affect MCP by reducing coagulation time and improving curd firmness; on the other hand, *A* and *E* variants of *CSN3* have shown negative effect on milk clotting [18,19]. Moreover, the A^1^ variant of *CSN2* improves rennet coagulation properties respect to A^2^ counterpart, [20], which has less influence on this technological property. Despite of several studies on the relation between MCP and genetic variants of *CSN1S1*, *CSN2* and *CSN3*, the evaluation of the effect of a particular locus can be confounded by the other causal loci in the genome. For this reason, studies on the influence of genetic factors affecting coagulation parameters of milk from different cattle breeds were focused on casein locus composite genotype rather than single genes [21,22,23].

The objective of this study was to analyse the genetic variability of *CSN1S1*, *CSN2* and *CSN3* genes in the Agerolese cattle breed and to evaluate the effect of single casein gene polymorphism and of casein composite genotype on milk quality traits and milk coagulation properties (MCP).

## 2. Materials and Methods

### 2.1. Ethical Statement

The Ethical Animal Care and Use Committee of University of Naples Federico II pre-approved all procedures used in this research study (Prot. Nr. PG/2019/0104896). 

### 2.2. Samples

Eighty-four Agerolese cows, enrolled in the Birth Register, and reared in 14 farms located in the production area of “Provolone del Monaco PDO” were involved in this study. All the animals received the same lactation diet according to the production disciplinary of “Provolone del Monaco PDO”. Milk and blood samples were collected from each animal for the genetic characterization at *CSN1S1*, *CSN2* and *CSN3* genes. All the samples were stored at 4 °C until analysis. Individual milk samples (50 mL) from sixty-three cows at the same lactation stage (70 to 200 d postpartum) were collected in the morning, during the spring, to evaluate the effect of casein composite genotype on quality and coagulation properties (MCP). All the milk samples were transported refrigerated and stored at 4 °C until analysis.

### 2.3. Genotyping by Ultra-Thin Layer Isoelectric Focusing on Polyacrylamide Gel (UTLIEF)

Individual genotyping at *CSN1S1* (A, B and C variants), *CSN2* (A^1^ and A^2^ variants), *CSN3* (A, B and C variants) was performed by Ultra-thin layer isoelectric focusing on polyacrylamide gel (UTLIEF). The isoelectrophoretic techniques is a recommended suitable analysis for routine typing of animals at the protein level. In a rapid, cheap and high-throughput way, it allows to detect α_s1_-, β- and κ-casein variants in individual or bulk milk samples, thus defining the casein genetic variability present in the bovine population. 

Individual whole bovine casein was prepared by acid precipitation of skim milk, followed by centrifugation at 2500× *g* for 15 min [24]. The casein obtained was rinsed twice with distilled water to eliminate whey, then freeze-dried and stored at −20 °C before use. Individual casein samples from individual milks (20 g/L) were dissolved in 9 M urea solution containing 2-mercaptoethanol (1 mL/L). UTLIEF was performed on a 2117 Multiphor II Apparatus (LKB, Bromma, Sweeden) at 10 °C using a Multitemp II (LKB, Bromma, Sweeden). Homemade polyacrylamide gel (265 × 125 × 0.25 mm; 4.5% T and 0.3% C) consisted of 7.2 M urea, 1% (*w/v*) glycerol and 1% (*v/v*), Ampholine (GE Healthcare Amersham Bioscience, Buckinghamshire, UK). To obtain the pH gradient 2.5–6.5 Ampholine 2.5–5, 4.5–5.4 and 4–6.5 in the ratio 1.6:1.4:1 (by vol) were mixed. TEMED and PER 0.04% (*v/v*) and 0.07% (*w/v*), respectively, were added to the gel, as activator and catalyst agents of polymerization. UTLIEF analysis consisted of three steps: pre-focusing set, 2000 V, 15 mA, 4 W, 30 min; sample focusing set, 2000 V, 15 mA, 4 W, 60 min; final focusing set, 3000 V, 5 mA, 20 W, 130 min. The gel was stained with Coomassie Brilliant Blue G-250 as described by Neuhoff et al. [25]. Agerolese cow’s milk samples were analyzed together with other bovine casein counterparts containing homozygous alleles respectively for A^1^ and A^2^ of β-CN, A and B of *κ-CN*, and B, C and A of α_s1_-CN for their genetic classification based on protein isoelectric point values. Protein variant identification in the milk samples was made by comparison with standard that consisted of purified genetic variants from bovine milk (Supplementary Figure S1).

### 2.4. Genotyping of CSN3 B Allele and CSN1S1 G Allele 

Since some electrophoretic profiles were unclear, a PCR-RFLP was performed to distinguish carriers of *CSN3* B variant. To this purpose, DNA was extracted from whole blood with Wizard® Genomic DNA purification kit (Promega, Madison, WI, USA). The DNA regions of the bovine *CSN3* gene spanning part of the exon 4 were amplified by iCycler (Bio-Rad, Hercules, CA, USA) using primers designed on the partial bovine genomic sequence (EMBL acc. no. X14908.1) as reported by Mitra et al. [26] (Table 1). PCR reaction mixture and thermal conditions were accomplished according to Ren et al. [27]. For genotyping, the digestion of 17 mL of each PCR amplification was accomplished with 10 U of *Hinf* I endonuclease (Promega, Madison, WI, USA) for 5 h at 37 °C following the supplier’s directions for experimental conditions.

In order to detect carriers of *CSN1S1* G allele, the DNA regions of the bovine *CSN1S1* gene spanning exon 19 and flanking regions were amplified by means of PCR using primers designed on the complete bovine *CSN1S1* genomic sequence (EMBL acc. no. X59856.2) (Supplementary Table S1). PCR reaction mixture and thermal conditions were accomplished using the method described by Rando et al. (1998) [28]. For both loci, all PCR amplifications were carried out using Bio-Rad T100 thermocycler (Bio-Rad, Hercules, CA, USA). PCR and digestion products were analysed directly by electrophoresis in 3% TBE agarose gel (Bio-Rad, Hercules, CA, USA) in 0.5X TBE buffer and stained with SYBR^®^ green nucleic acid stain (Lonza - Rockland – ME - USA).

### 2.5. Milk Chemical Composition and MCP

Milk chemical composition and coagulation properties were evaluated at the Laboratory of milk analyses of Italian Breeders Association (AIA). Milk samples were transported refrigerated (4 °C) and analysed within 12 h from collection. Total solids, solid-non-fat, fat, protein, casein, lactose, ash, RCT, k_20_, a_30_ were measured using MilkoScan FT 6000 milk analyzer (Foss Electric A/S, Hillerød, Denmark) according to ISO 9622 IDF 141C: 2013. MCPs were predicted using mid-infrared spectroscopy models developed by De Marchi et al. [29,30].

All samples had somatic cell count (SCC) below 500,000 cells/mL (269,000 ± 210,000).

### 2.6. Statistical Analysis 

Correlation among composite genotypes, chemical composition and MCP values of milk were performed only for composite genotypes with a minimum of nine animals. Allele frequencies were calculated by simple allele counting [31]. Possible deviations of genotypic frequencies from expectations under Hardy–Weinberg equilibrium were tested by a chi-square test. Some population genetic indices, such as: gene heterozygosity (He), gene homozygosity (Ho), effective allele numbers (Ne) and Fixation Index (FIS) were performed by POPGENE32 software version 1.32 [32]. Finally, polymorphism information content (PIC) was calculated according to Botstein et al. [33]. The variables of this study were numeric and continuous. All production data were tested for normality and homoscedasticity. Normality test were applied using Shapiro–Wilk criterion [34]. All the considered variables did not deviate from a normal distribution (*p* > 0.05). Moreover, homoscedasticity was tested with Levene’s Test [35] that evidenced a homoscedastic distribution of data (*p* > 0.05). A statistical analysis was carried out to estimate the effect of single αs1- β- and ĸ-casein genotypes and composite genotypes on milk composition traits and MCP. The GLM procedure, implemented with SAS software (Ver. 9.2) [36], included the single loci genotypes, the composite genotype and the parity (first, second and third and later parities) as fixed effects. Composite genotypes with less than nine individuals were excluded from the statistical analysis. If more than two groups were compared, the Bonferroni test was used for multiple testing. The values were considered significant at *p* < 0.05 and presented as least squares means.

## 3. Results

### 3.1. Gene Frequency and Composite Genotypes

Table 1 shows the allelic and genotypic frequencies at *CSN1S1*, *CSN2* and *CSN3.* The Agerolese population was found to be polymorphic at α_s1_-casein encoding locus: in particular, 61 individuals out of 84 were genotyped as BB; 22 as BC and only one cow was homozygous CC. The frequencies of B and C alleles were 0.857 and 0.143, respectively. The expected genotype frequencies, which were calculated according to the Hardy–Weinberg equilibrium, were 73.40% (BB), 24.64% (BC), and 1.96% (CC). The calculated *χ^2^* value was 0.35 (d.f. = 1), indicating Hardy–Weinberg equilibrium in the population (*p* = 0.55). No carriers of the rare G allele were detected. As regard *CSN2*, the most frequent genotypes in the investigated population were A^2^A^2^ and A^1^A^2^ (48.8% and 35.71%, respectively), which were followed by A^1^A^1^ and A^1^B (9.53% and 5.95%, respectively). The allele frequencies at *CSN2* locus were 0.303 (allele A^1^), 0.667 (allele A^2^) and 0.030 (allele B). The comparison between observed and expected genotype frequencies revealed no Hardy-Weinberg equilibrium in the population for this locus (*χ^2^* = 12.23; *p* < 0.01; d.f. = 3). As regard *CSN3* locus, 54 Agerolese cows (64.29%) were genotyped as AB, 23 (27.38%) as BB and 7 (8.33%) as AA. Thus, the allelic frequencies were 0.405 and 0.595 for A and B alleles, respectively. The distribution of genotypes deviated significantly from Hardy-Weinberg expectations (*χ^2^* = 9.05; *p* < 0.01; d.f. = 1).

Table 2 also illustrates the calculated values of the genetic indices, such as Ho, He, Ne, PIC and FIS for the considered loci. FIS is a measurement of the deviation of genotypic frequencies from panmictic frequencies in terms of heterozygous deficiency or excess. Negative FIS values indicate heterozygote excess, while positive values indicate heterozygote deficiency when compared with Hardy-Weinberg equilibrium expectations. In the Agerolese breed, an excess of heterozygosity at *CSN3* locus was found (FIS = −0.334). PIC is a parameter indicative of the degree of informativeness of a certain marker. The PIC value may range from 0 to 1. In the Agerolese population, PIC values were 0.215, 0.455 and 0.366 for *CSN1S1, CSN2* and *CSN3*, respectively. According to the classification of PIC (low polymorphism if the PIC value < 0.25, median if 0.25 < PIC value < 0.50 and high if the PIC value > 0.50), the examined population seemed to have a low genetic diversity at *CSN1S1* locus; *CSN2* and *CSN3* loci result more informative showing an intermediate polymorphism level. These results were also confirmed based on the calculated Ne values, which indicated a good level of genetic variability in this breed. 

As regard the composite genotypes in the analysed Agerolese cows, 19 different combinations have been identified (Supplementary Table S2). 

### 3.2. Analysis of Milk Samples

The chemical composition and MCP values (Mean and SD) of the Agerolese cattle milk according to the genotypes at *CSN1S1*, *CSN2* and *CSN3* loci are shown in Table 3. None of the analysed milk samples was non-coagulating.

Regarding *CSN1S1*, the genotype BB showed a statistically significant higher fat content (3.74 ± 0.06) and lower k_20_ (6.03 ± 0.16) than the genotype BC (3.48 ± 0.15; 7.75 ± 0.41). However, since the BC genotype carriers found within this work are very few (*n* = 4), and no one showed a CC genotype on *CSN3*, it was not possible to establish with certainty the effect of BC genotype on milk production traits in Agerolese cattle. As regard to *CSN2*, the A^2^A^2^ genotype showed a statistically significant higher content of protein, total solids and solid-non-fat (3.49 ± 0.06; 12.73 ± 0.14; 8.97 ± 0.06 respectively) than the A^1^A^1^ genotype (3.21 ± 0.11; 12.01 ± 0.28; 8.64 ± 0.12). A^1^A^2^ genotype showed intermediate values for the same parameters (3.43 ± 0.05; 12.72 ± 0.13; 8.90 ± 0.05). This is in line with previous studies [37]. A similar condition occurred for MCP; in fact, RCT and k_20_ were faster in a statistically significant way for the A^2^A^2^ genotype and a_30_ value was higher (13.50 ± 0.52; 5.76 ± 0.25; 24.93 ± 1.10, respectively). Contrary to prior research results [18,38], the A^2^A^2^ genotype in Agerolese cattle was associated with better MCP values. Finally, a statistically significant higher fat content (3.76 ± 0.09 vs. 3.56 ± 0.07) and shorter curd firming time (k_20_) (5.81 ± 0.25 vs. 6.40 ± 0.20) were observed in BB rather than AB genotype at *CSN3*. These findings are in agreement with previously studies [18].

Considering the effects of the composite genotypes observed by the three loci analyzed on milk chemical composition and MCP values (Table 4), BBA^1^A^2^AB composite genotype showed a statistically significant higher fat content (3.93 ± 0.10) than the other ones, while for BBA^1^A^2^BB composite genotype there was a statistically increased lactose content (4.77 ± 0.07). Regarding the protein content, it was similar amongst the four composite genotypes analysed. No statistically significant differences were observed for all the other investigated chemical parameters. 

## 4. Discussion 

The analysis of polymorphisms of genes associated to productive traits in a livestock breed is the first step necessary for the improvement of its profitability. It should not be forgotten that often, in the same species, with same environmental effects, as the breeds varies, a certain level of productivity can be associated with different polymorphisms in the same genes or with completely different genes [18,38,39]. This is the reason why we need not only to verify the genotype of a livestock breed of interest but also to correlate it with productions. Native breeds are of great interest because they are important reservoirs of genetic variability, which may be targeted by conservation efforts. Moreover, they might possess unique genetic variation that can affect the milk composition and quality, potentially resulting in distinct milk characteristics that could be exploited in niche dairy products [40]. In this study, 84 Agerolese cattle, representing about 30% of the entire population (285 animals enrolled in the Birth Register) have been analysed for casein composite genotypes and for MCPs.

Genotyping results for the three casein genes analysed showed that in Agerolese cattle are spread only the most common alleles of these genes; interestingly 19 different composite genotypes have been observed indicating a good variability at this level. According to the electrophoretic patterns (UTLIEF) of the milk caseins observed in all the analysed animals, there are no new variants to report in this breed. Analysing *CSN1S1* gene, the alleles found and their frequencies confirmed what has been previously observed in other cattle breeds, namely that C allele is not common in *Bos taurus*, except for few breeds like Jersey, Guernsey, Normande, Italian Brown, Reggiana, Modenese and Normande [41]. In particular, in Jersey its frequency is very high and it might indicate a positive effect on fat and protein content [42]. This does not occur in Agerolese cattle in which milk from heterozygous individuals showed a lower content of both nutrients (Table 2) when compared with homozygous BB animals. The very low number of CC animals in the sample analysed does not allow stating definitively the effects of *CSN1S1* C variant in Agerolese cattle milk productions. Interestingly, the allele frequencies were the same observed in Brown Swiss [42], which contributed to the constitution of current Agerolese cattle.

As regard *CSN2*, FIS value indicates a heterozygote deficiency that is due to the greater frequency of A^2^ allele. This is the preferred protein variant for potentially improving human health and, contrary to what has been observed in other breeds [20], A^2^A^2^ genotype in Agerolese cattle seems to be associated with better milk parameters, in particular with respect to MCP. Its prevalence in the population may be due to an unintentional selection performed by the breeders, which were mainly interested in milk for cheese production. Moreover, β-casein protein encoded by A^1^ allele is considered a risk factor for human ischemic heart disease, arteriosclerosis, Type 1 diabetes, sudden infant death syndrome and autism [43]; thus, Agerolese cattle milk products may have healthy properties which would be interesting to investigate. At *CSN3* gene *locus*, a very low number of animals carry the AA genotype; this is probably due to the worst MCPs that are typically associated with this genotype [37]. Finally, the analysis of population indexes upholds what has already been observed in terms of overall genetic variability found in other loci studied in the same breed [2,3].

When considering the number of different composite genotypes found (*n* = 19) a surprisingly high variability is observed. This is even more evident when comparing this data with that of other breeds either native or not like German Black Pied Cattle, Swedish Red, Swedish Mountain and Swedish Red Polled [40,42], Italian Holstein [16], Danish Holstein and Danish Jersey [44]. In fact, if considering composite genotypes deriving from combinations of only the alleles found in Agerolese cattle, not one of them showed to carry so high a number of different composite genotypes. With respect to the frequencies of the composite genotypes BBA^1^A^2^AB and BBA^2^A^2^AB are the most common (19.05%), while BBA^1^A^1^AA and BBA^2^A^2^AA are among the least common (1.19%). This is congruent with the production address of Agerolese cattle milk, in fact, according to Perna et al. [16], BBA^2^A^2^AB composite genotype is positively associated with a high yield of *pasta filata* cheese while BBA^1^A^1^AA and BBA^2^A^2^AA are negatively associated to this production.

Fat, protein, casein and lactose content values of Agerolese cattle milk were 3.68 ± 0.83, 3.45 ± 0.50, 2.67 ± 0.41 and 4.67 ± 0.36, respectively. These values are similar to those previously reported by Matassino et al. [45] indicating the absence of population change characteristics in the last twelve years. This is normal considering that the Agerolese population is constituted by a very small number of individuals and no selection for any traits has been carried so far on. This could also explain the normality of the data distribution and the residuals homoscedasticity. In fact, selection pressure may be the reason why in same cases an erosion of the left tail of data distribution may be observed. 

Considering MCP values, the best RCT was observed for milks of BBA^2^A^2^BB composite genotype, while optimal k_20_ value occurred for milks of the BBA^2^A^2^AB composite genotype. These findings are in agreement with those reported for the Italian Holstein cow [16]. 

## 5. Conclusions

The study of gene polymorphisms at loci of productive interest allows to evaluate how a breed is suitable for the productions for which it is reared. The analysis of the allele variants of casein genes in the Agerolese cattle shows a low genetic variability at *CSN1S1*, *CSN2* and *CSN3*. Among the genotypes identified, the *CSN2* A^2^A^2^ is the one associated with better milk characteristics meaning that selection plans with the aim to improve productions in this breed are conceivable. In particular, the wide spread of the A^2^ variant in the population allows to plan future studies aimed to increase the number of the analysed animals to confirm these results and at evaluating the effects of this variant on the quality of the Provolone del Monaco PDO cheese from a nutraceutical point of view.

Despite the low variability found in all the loci when considered alone, a high variability of the composite genotypes (*n* = 19) has been observed, with a prevalence of those (BBA^1^A^2^AB and BBA^2^A^2^AB) positively associated with the production of milk addressed to cheese manufacture and in particular, to the *pasta filata* type. These findings support the hypothesis that Agerolese Cattle is an important reservoir of genetic variability the conservation of which today represents one of the aims pursued worldwide in the zootechnical field. Moreover, these data underline the importance of implementing plans that encourage their rearing throughout the territory, limiting the erosion process it is undergoing. 

## Figures and Tables

**Table 1:** Observed and expected numbers and percentages (in brackets) of genotypes at *CSN1S1*, *CSN2* and *CSN3* loci, allele frequencies and Hardy-Weinberg Equilibrium (HWE) in the sample of Agerolese cows.

**Casein Locus**		**Genotype Frequency**						**Allele Frequency**			***χ^2^*** **HWE**
*CSN1S1*		**BB**	**BC**	**CC**				**B**	**C**		
	Obs.	61 (72.62%)	22 (26.19%)	1 (1.19%)				0.857	0.143		0.35 *p* = 0.55 (d.f. = 1)
									
	Exp.	61.65 (73.40%)	20.70 (24.64%)	1.65 (1.96%)							
*CSN2*		A1A1	A1A2	A2A2	A^1^B	A^2^B	BB	A1	A2	B	
	Obs.	8 (9.53%)	30 (35.71%)	41 (48.8%)	5 (5.95%)	-	-	0.303	0.667	0.030	12.23 *p* < 0.01 (d.f. = 3)
									
	Exp.	7.64 (9.10%)	34.20 (40.71%)	37.22 (44.31%)	1.53 (1.82%)	3.35 (3.99%)	0.06 (0.07%)				
*CSN3*		AA	AB	BB				A	B		
	Obs.	7 (8.33%)	54 (64.29%)	23 (27.38%)				0.405	0.595		9.05 *p* < 0.01 (d.f. = 1)
									
	Exp.	13.64 (16.24%)	40.72 (48.48%)	29.64 (35.28%)							

**Table 2:** Population genetic indices for the three considered loci in the sample of Agerolese cows.

Parameter	*CSN1S1*	*CSN2*	*CSN3*
Gene homozygosity (Ho)	0.738	0.583	0.357
Gene heterozygosity (He)	0.262	0.417	0.643
Effective allele number (Ne)	1.324	1.861	1.930
Polymorphism Information Content (PIC)	0.215	0.455	0.366
Fixation Index (FIS)	−0.069	0.099	−0.334

**Table 3:** Chemical composition (g/100 g) and MCP of Agerolese cattle milk according to the genotype at *CSN1S1*, *CSN2* and *CSN3*.

	*CSN1S1*		*CSN2*			*CSN3*	
Trait	BB *n* = 51	BC *n* = 4	A^1^A^1^ *n* = 8	A^1^A^2^ *n* = 25	A^2^A^2^ *n* = 20	AB *n* = 29	BB *n* = 19
Total solids	12.66 ± 0.09	12.25 ± 0.24	12.01^a^ ± 0.28	12.72^b^ ± 0.13	12.73^b^ ± 0.14	12.58 ± 0.12	12.51 ± 0.14
Solid-non-fat	8.91 ± 0.04	8.71 ± 0.10	8.64^a^ ± 0.12	8.90^b^ ± 0.05	8.97^b^ ± 0.06	8.87 ± 0.05	8.85 ± 0.06
Fat	3.74^a^ ± 0.06	3.48^b^ ± 0.15	3.66 ± 0.17	3.81 ± 0.08	3.67 ± 0.09	3.56 ± 0.07	3.76 ± 0.09
Protein	3.44 ± 0.04	3.23 ± 0.10	3.21^a^ ± 0.11	3.43 ± 0.05	3.49^b^ ± 0.06	3.41 ± 0.05	3.42 ± 0.06
Casein	2.66 ± 0.03	2.55 ± 0.08	2.46^a^ ± 0.09	2.65^b^ ± 0.04	2.71^b^ ± 0.05	2.64 ± 0.04	2.64 ± 0.05
Lactose	4.69 ± 0.03	4.63 ± 0.07	4.69 ± 0.08	4.68 ± 0.04	4.69 ± 0.04	4.68 ± 0.03	4.65 ± 0.04
Ash	0.78 ± 0.01	0.76 ± 0.02	0.74 ± 0.02	0.79 ± 0.01	0.79 ± 0.01	0.78 ± 0.01	0.78 ± 0.01
RCT (min)	14.31 ± 0.34	15.49 ± 0.88	15.80^a^ ± 1.00	14.80 ± 0.46	13.50^a^ ± 0.52	14.82 ± 0.42	13.77 ± 0.52
k_20_ (min)	6.03^A^ ± 0.16	7.75^B^ ± 0.41	7.55^A,a^ ± 0.48	6.43^b^ ± 0.21	5.76^B^ ± 0.25	6.40^a^ ± 0.20	5.81^b^ ± 0.25
a_30_ (mm)	23.01 ± 0.48	25.17 ± 1.24	21.75^a^ ± 1.46	23.22 ± 0.66	24.93^b^ ± 1.10	23.53 ± 0.61	22.45 ± 0.76

A,B: *p* < 0.01; a,b: *p* < 0.05.

**Table 4:** Chemical composition (g/100 g) and MCP of Agerolese cattle milk according to the composite genotype.

Trait	BBA^1^A^2^AB *n* = 16			BBA^1^A^2^BB *n* = 9			BBA^2^A^2^AB *n* = 9			BBA^2^A^2^BB *n* = 10		
Total solids	12.70	±	0.16	12.66	±	0.21	12.49	±	0.21	12.50	±	0.20
Solids-non-fat	8.82	±	0.07	8.97	±	0.09	9.03^a^	±	0.09	8.78^b^	±	0.09
Fat	3.93^A,a^	±	0.10	3.61^b^	±	0.13	3.40^B^	±	0.13	3.62^b^	±	0.12
Protein	3.40	±	0.07	3.43	±	0.09	3.48	±	0.09	3.43	±	0.08
Casein	2.62	±	0.05	2.67	±	0.07	2.72	±	0.07	2.64	±	0.06
Lactose	4.62	±	0.05	4.77^a^	±	0.07	4.74^a^	±	0.07	4.54^b^	±	0.06
Ash	0.79	±	0.02	0.77	±	0.02	0.80	±	0.02	0.80	±	0.02
RCT (min)	14.94^a^	±	0.64	14.08	±	0.85	14.84	±	0.85	12.62^b^	±	0.81
k_20_ (min)	6.65^a^	±	0.29	5.80	±	0.39	5.40^b^	±	0.39	5.84	±	0.37
a_30_ (mm)	23.51	±	0.87	21.24	±	1.16	23.35	±	1.16	23.72	±	1.10

A,B: *p* < 0.01; a,b: *p* < 0.05.

## Supplementary Materials

The following are available online at https://www.mdpi.com/2076-2615/10/5/892/s1. **Supplementary Figure S1.** The figure shows the 13 most representative electrophoretic profiles of 84 individual milk samples analysed. On the top of the lane is reported the genotype of *CSN1S1.* The arrows highlight typical bands of main variants of *CSN2* (A_1_, A_2_ and B variants) and *CSN3* (A and B variants). **Supplementary Table S1.** Oligonucleotide primers sequence and positions. **Supplementary Table S2.** Frequencies of *CSN1S1, CSN2* and *CSN3* composite genotypes.
