# Supplementary material for: Influence of the Casein Composite Genotype on Milk Quality and Coagulation Properties in the Endangered Agerolese Cattle Breed
Source: Animals (Basel). 2020 May 20;10(5):892. doi: 10.3390/ani10050892 (PMC7278449; doi:10.3390/ani10050892)
Supplement: Supplementary file 1 [file animals-10-00892-s001.pdf]

Supplementary Material

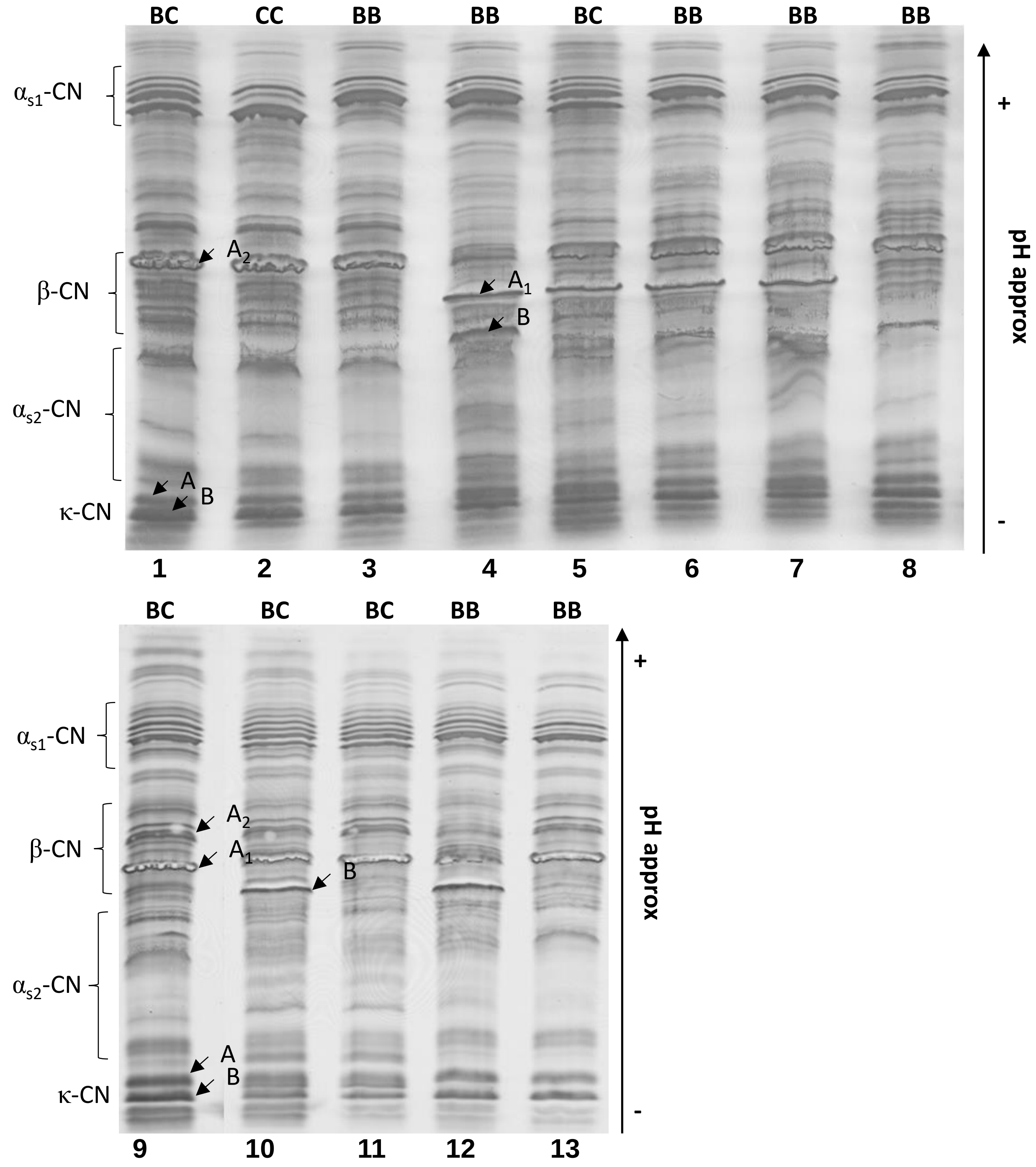

Supplementary Figure 1. The figure shows the 13 most representative electrophoretic profiles of the analysed 84 individual milk samples analysis. On the top of the lane is reported the genotype of *CSN1S1.* The arrows highlight typical bands of main variants of *CSN2* (A_1, A_2 and B variants) and *CSN3* (A and B variants).

Supplementary Table 1. Oligonucleotide primers sequence and positions.

Locus		Position nt	Primers sequence (5′-3′)		EMBL
CSN3	Exon 4	5189-5213	Forward	CACGTCACCCACACCCACATTTATC	X14908.1
		Complementary to: 5567-5542	Reverse	TAATTAGCCCATTTCGCCTTCTCTGT	
CSN1S1	Exon 19	27587 - 27604	Forward	CATGCAGCATAACTAACC	X59856.2
		Complementary to: 28269 - 28252	Reverse	TACTACACTGCTGTTCTC	

Supplementary Table 2. Frequencies of *CSN1S1, CSN2* and *CSN3* composite genotypes.

Composite genotype	%
BBA^{1}A^{2}AB	19.05
BBA^{2}A^{2}AB	19.05
BCA^{2}A^{2}AB	13.11
BBA^{2}A^{2}BB	11.90
BBA^{1}A^{2}BB	10.71
BCA^{1}A^{1}AB	3.57
BBA^{1}A^{1}AB / BBA^{1}A^{2}AA / BBA^{1}BAB / BCA^{1}A^{2}AB / BCA^{1}BBB / BCA^{2}A^{2}AA	2.38
BBA^{1}A^{1}AA / BBA^{1}A^{1}AB / BBA^{2}A^{2}AA / BBA^{1}BBB / BCA^{1}A^{1}AA / BCA^{1}A^{2}BB / CCA^{2}A^{2}AB	1.19

21
